# Prevalence and Trends in Percutaneous Endoscopic Gastrostomy Placement: Results From a 10-Year, Nationwide Analysis

**DOI:** 10.3389/fnut.2022.906409

**Published:** 2022-05-30

**Authors:** Marcin Folwarski, Stanislaw Klek, Michał Brzeziński, Agnieszka Szlagatys-Sidorkiewicz, Adam Wyszomirski, Jarosław Meyer-Szary, Karolina Skonieczna-Żydecka

**Affiliations:** ^1^Department of Clinical Nutrition and Dietetics, Medical University of Gdańsk, Gdańsk, Poland; ^2^General Surgery Department, Home Enteral and Parenteral Nutrition Unit, Nicolaus Copernicus Hospital, Gdańsk, Poland; ^3^Surgical Oncology Clinic, Maria Skłodowska-Curie National Cancer Institute, Kraków, Poland; ^4^Department of Pediatrics, Gastroenterology, Allergology, and Nutrition, Medical University of Gdańsk, Gdańsk, Poland; ^5^Department of Adult Neurology, Faculty of Medicine, Medical University of Gdańsk, Gdańsk, Poland; ^6^Department of Pediatric Cardiology and Congenital Heart Defects, Medical University of Gdańsk, Gdańsk, Poland; ^7^Department of Biochemical Sciences, Pomeranian Medical University in Szczecin, Szczecin, Poland

**Keywords:** PEG, gastrostomy, enteral nutrition, home enteral nutrition, head and neck cancer

## Abstract

**Background:**

Percutaneous endoscopic gastrostomy (PEG) is the most commonly used access for long-term enteral nutrition. Only a few studies report the prevalence and epidemiology of PEG placements. No previous data concentrated on the healthcare system issues influencing the qualification rates and professional nutritional support for individuals with PEG.

**Methods:**

We conducted a retrospective nationwide analysis of PEG placements in Poland from 2010 to 2020. The central data on ICD-10 coding of adult patients with PEG reported to the insurance company were used for the analysis of general and regional prevalence, age, and primary and secondary diseases. Rates of patients with home enteral nutrition (HEN) were calculated with a special focus on patients with cancer. A secondary aim was to determine the causes of regional disparities among administrative regions.

**Results:**

A total number of 90,182 PEGs were placed during the observation period. The number was increasing each year with statistical significance. Malnutrition, dysphagia, and cardiorespiratory/metabolic diseases were the most frequently reported primary diseases. A total of 11.98% of all patients were diagnosed with cancer; 49.9% of oncological patients suffered from head and neck cancer (HNC) and 19.9% from esophageal cancer. In total, 6.61% of HNC and 27.46% of patients with esophageal cancer from the Polish National Cancer Registry (NCR) had PEG. The rates of patients in more advanced ages (65–74 and over 85 years) were growing and decreased in younger groups (18–24, 45–54, and 55–64 years). Overall, 27.6% of all (11.86% of cancer) patients with PEG were reimbursed HEN. A high number of patients in nursing care facilities, lower education of citizens, and lower number of hospital beds were associated with more PEG insertions in the administrative regions.

**Conclusion:**

The number of PEG placements has been increasing, particularly in the elderly. Systemic solutions must be found to address the problems of regional disparities in PEG’s prevalence as well as the lack of inclusion criteria for nutritional support.

## Introduction

According to the guidelines of the European Society of Clinical Nutrition (ESPEN), percutaneous endoscopic gastrostomy (PEG) tube placement is indicated for patients requiring long-term enteral nutritional support (1). Since the first publication on endoscopic PEG in 1980, this method remains the most common worldwide (2). According to epidemiological data, the number of patients receiving home enteral nutrition (HEN) is increasing (3, 4). Nevertheless, there are different strategies for enteral nutritional routes. Gastrostomy is the most prevalent access in HEN in Poland (77% of patients) (5). However, other European studies show that nasogastric tubes (NGT) are more commonly used. In the Italian HEN population, 60% of neurological patients, 36% of patients with head and neck (HNC) cancer, and 23% of patients with abdominal cancer were on NGT (3). In studies of the National Registry of Patient-Spanish Society for Parenteral and Enteral Nutrition (NADYA-SENPE) Group, PEG was only applied in 6.8–10% of HEN patients (6, 7).

The prevalence of PEG tube placement depends on various factors. Developing support for early diagnosis and treatment of swallowing problems may delay the need for tube feeding (8). On the other hand, a lack of awareness of the consequences of malnutrition results in insufficient numbers of patients on nutritional support. Additionally, the attitude of healthcare professionals toward PEG depends on the cultural and geographical settings around the world. A systematic review of Jaafar et al. showed that medical staff in North American and European studies were more positive about PEG than Asian and Turkish. Inadequate knowledge and skills, health system and financial resources (availability of public funding), family influences, and cultural or religious beliefs were differentiating factors. Nutrition and the end-of-life issues were also perceived differently (especially in Asian cultures) (9). There is a lack of clear evidence on the benefit of the survival and functioning of elderly patients with dementia and the indications for gastrostomy placement are a matter of debate (10).

For countries with large numbers of uninsured patients or with complex healthcare systems, nationwide studies are challenging. In Poland, there is one public, national healthcare provider (National Health Fund—NHF), which gathers all data on publicly financed medical procedures. About 95% of citizens of Poland are covered by NHF financing. Since 2007, home enteral nutrition is reimbursed exclusively by NHF, which enables scientific and epidemiological analyses. In our study, we aimed to describe the prevalence of gastrostomy placement by analyzing the trends and regional disparities in long-term observation.

## Materials and Methods

We conducted a retrospective nationwide analysis of medical records reported to the National Health Fund between 2010 and 2020. All adult patients with PEG were included in the study. Collected information was identified with the International Classification of Diseases (ICD-10) and the Diagnosis and Procedure Codes Classification (ICD-9). Furthermore, 43.11 and 43.19 ICD-9 codes for gastrostomies were selected. Data were anonymized so that no individual patient could be identified. The study was conducted in accordance with the ethical standards of the Helsinki Declaration.

Prevalence data for primary and secondary diseases, age, and gender were collected for all administrative regions in Poland. Numbers and rates of patients with gastrostomies in the HEN program were analyzed (available from 2010 to 2019). Trend analysis was calculated for prevalence data (primary disease and age groups) and HEN qualifications. In the subgroup of patients with cancer, disease-specific analyses on the rates of PEG placements were performed. Data on all registered patients with cancer in Poland (available from 2010 to 2018) were obtained from the open-access Polish database of the National Cancer Registry (NCR) (11). Diagnoses were grouped into categories: non-cancer gastro-intestinal (NON-CANCER-GI), cardio-respiratory and metabolic (CRM), neurology (NEURO), malnutrition and dysphagia (MD), cancer (CANCER), and other (OTHER). Detailed information on specific diagnoses assigned to categories is presented in Table 1.

**TABLE 1:** Detailed data on the primary diagnosis of patients with gastrostomy.

Primary diagnosis	% of category		% of all
Non-cancer GI			
Gastric/duodenal/esophageal inflammation, ulcerations, or GI reflux disease	74.04%		5.54%
IBD and other intestinal diseases	3.99%		0.30%
Liver and pancreatic non-cancer diseases	7.19%		0.54%
Paralytic ileus	4.09%		0.31%
Hereditary diseases of the GI	0.55%		0.04%
Other non-cancer GI diseases	10.14%		0.76%
Other			
Attention to artificial opening	41.57%		5.40%
Trauma	23.89%		3.10%
Postoperative disorders	0.68%		0.09%
Shock	1.48%		0.19%
Thrombotic disease	0.47%		0.06%
Intoxication	0.32%		0.04%
Burns	0.30%		0.04%
Obesity	0.14%		0.02%
Chronic wound care	5.63%		0.73%
Other, not specified	15.86%		2.06%
Infections not specified	9.67%		1.26%
Cardio-resp. and metabolic			
Other respiratory diseases	43.20%		8.36%
Cardiologic/heart failure	33.80%		6.54%
Pneumoniae	12.46%		2.41%
Kidney and urinary tract diseases	6.45%		1.25%
Dehydration	1.21%		0.23%
Anemia	1.08%		0.21%
Diabetes	0.98%		0.19%
Pulmonary thrombosis	0.70%		0.14%
Cystic fibrosis	0.13%		0.03%
Neurology			
Stroke	72.76%		8.93%
Neurodegenerative/dementia	4.82%		0.59%
Spinal muscular atrophy (SMA)	5.84%		0.72%
Neuro-muscular diseases	4.05%		0.50%
Epilepsy	2.29%		0.28%
Neural infections	1.12%		0.14%
Mental disorders	1.41%		0.17%
Multiple sclerosis	0.76%		0.09%
Cerebral palsy	0.32%		0.04%
Other Hereditary neural diseases	0.72%		0.09%
Huntington disease	0.21%		0.03%
Other neurological	5.69%		0.70%
Malnutrition/Dysphagia			
Dysphagia	88.11%		32.81%
Malnutrition	11.89%		4.43%
Cancer	PD	PD&SD	% of all
Head and neck	29.72%	49.9%	3.16%
Respiratory and mediastinum	6.42%	5.3%	0.68%
Esophageal	31.77%	19.9%	3.38%
Gastric	15.50%	7.4%	1.65%
Other GI (liver, pancreatic, intestinal)	12.82%	9.7%	1.37%
Other/not specified	3.77%	7.8%	0.40%

*Non-cancer GI, Non-cancer gastro-intestinal; IBD, Inflammatory bowel diseases; PD, primary diagnosis; SD, Secondary diagnosis.*

## Secondary Aims of the Study

The additional aim was to analyze factors influencing the disparities between administrative regions (voivodeships) of Poland in the rates of gastrostomy placement. Demographics (general population, citizens of cities, and villages), healthcare (number of registered physician practices and practicing physicians, hospital beds, and patients in-home care facilities), and education (rates of citizens with high education in urban and rural areas) data were compared yearly and regionally. These data were obtained from an open-source database of the Polish Department of Statistics (12).

### Statistical Analysis

Qualitative variables were shown as counts with percentages. We built linear regression models to verify possible trends across years. During this process, we treated our dataset as a sample rather than a population. Results from linear regression were expressed as beta coefficients with 95% confidence intervals (*CI*s). The two-tailed tests were carried out at a significance level of 0.05. Statistical analysis was performed using the R statistical package (version 3.6.3.).

## Results

### Indications for Percutaneous Endoscopic Gastrostomy

**FIGURE 1:**
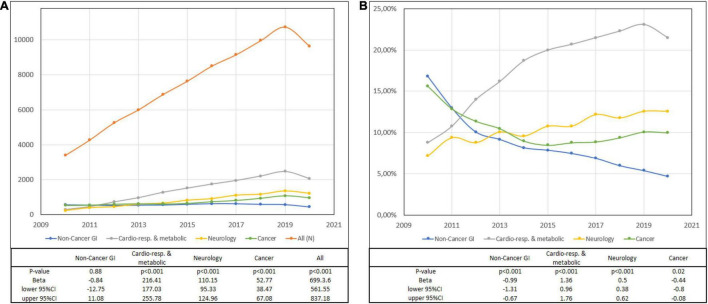
Primary diagnosis and trends. **(A)** Number of percutaneous endoscopic gastrostomies (PEGs) and **(B)** the percentage of PEGs with a primary diagnosis.

A total of 90,182 gastrostomies were placed from 2010 to 2020 (43.38% women and 56.62% men) with 81,591 primary disease records. The median number of PEGs in Poland was 8,413 per year with a significant growth in the observation period (***p <*** 0.001). MD was frequently reported as the primary disease, while CRM as secondary disease. Upper GI ulceration/inflammation or gastroesophageal reflux was the most common diagnosis from NON-CANCER-GI, accounting for nearly two-thirds of the patients in this group. In the OTHER group, more than 41% of patients were qualified for PEG due to stoma care (attention to artificial opening ICD-10 code) and 23.9% trauma. CRM consisted mainly of respiratory diseases (43.2%), cardiology and heart failure (33.8%), and pneumonia (12.46%). The dominating disease in the NEURO category was stroke (72%) and only 4.82% were patients with neurodegenerative disorders and dementia (Table 1). A 2.02-fold increase from 2010 to 2019 (1.8-fold from 2010 to 2020) with primary diagnosis (10.65% of all patients) and a 2.5-fold increase from 2010 to 2019 (1.5-fold from 2010 to 2020) with primary or secondary diagnosis (11.98% of all patients) of patients with CANCER was observed. Trend analysis showed growing numbers of PEGs in all primary diagnosis groups except for NON-CANCER-GI diseases. The rates of patients with diseases categorized as NON-CANCER-GI, CANCER, and OTHER decreased but CRM, NEURO, and MD increased statistically significant (Figure 1; Supplementary Table 3). There were 169,267 primary and secondary diagnosis records with the most common being CRM, MD, and NEURO (Table 2).

**TABLE 2:** Primary and secondary diagnosis.

Year	Non-cancer GI (N;%)	Other (N;%)	Cardio-resp. and metabolic (N;%)	Neurology (N;%)	Malnutrition/Dysphagia (N;%)	Cancer (N;%)	Overall (N;%)
2010	767; 12.39%	989; 15.97%	1353; 21.85%	747; 12.07%	1284; 20.74%	1051; 16.98%	6191; 100%
2011	778; 9.37%	1026; 12.36%	2117; 25.5%	1134; 13.66%	2011; 24.23%	1235; 14.88%	8301; 100%
2012	750; 7.12%	1249; 11.85%	3049; 28.93%	1490; 14.14%	2605; 24.72%	1395; 13.24%	10538; 100%
2013	901; 7.06%	1405; 11.01%	4039; 31.66%	1810; 14.19%	3012; 23.61%	1589; 12.46%	12756; 100%
2014	892; 5.99%	1512; 10.15%	5093; 34.2%	2157; 14.48%	3560; 23.91%	1678; 11.27%	14892; 100%
2015	1026; 6.12%	1737; 10.36%	5801; 34.61%	2608; 15.56%	3890; 23.21%	1700; 10.14%	16762; 100%
2016	1143; 5.94%	1852; 9.63%	6765; 35.17%	3001; 15.6%	4363; 22.68%	2112; 10.98%	19236; 100%
2017	1094; 5.3%	2103; 10.18%	7438; 36.01%	3269; 15.83%	4554; 22.05%	2198; 10.64%	20656; 100%
2018	1079; 4.77%	2192; 9.69%	8232; 36.38%	3492; 15.43%	5121; 22.63%	2512; 11.1%	22628; 100%
2019	1124; 4.56%	2368; 9.6%	9295; 37.68%	3916; 15.87%	5325; 21.58%	2642; 10.71%	24670; 100%
2020	514; 4.07%	1284; 10.16%	6042; 47.81%	2204; 17.44%	999; 7.91%	1594; 12.61%	12637; 100%
Overall	10068; 5.95%	17717; 10.47%	59224; 34.99%	25828; 15.26%	36724; 21.7%	19706; 11.64%	169267; 100%

### Age Distribution

The number of patients in all age groups (except for individuals between 18 and 24 years) increased during the observation time. Growing rates of patients aged 65–74 and over 85 (trend *p* < 0.001) and decreasing percentages of individuals aged 18–24, 45–54, and 55–64 (Supplementary Table 1) were observed.

### Patients With Percutaneous Endoscopic Gastrostomy on Home Enteral Nutrition

**FIGURE 2:**
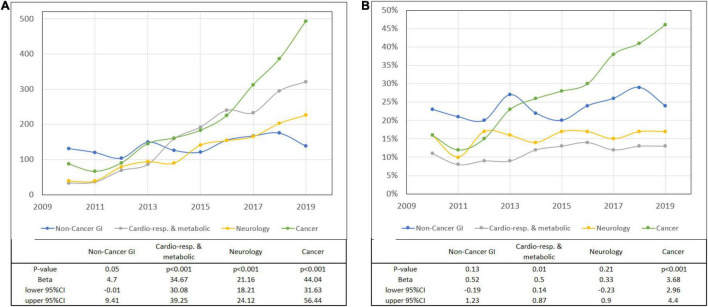
Patients with gastrostomies on home enteral nutrition (HEN) (primary diagnosis). **(A)** Number and **(B)** the percentage of PEGs according to the primary diagnosis.

Trend analysis showed significant growth in the numbers of patients with gastrostomies qualified for the HEN program with primary disease categories of CRM, MD, and OTHER. The numbers of patients in CANCER, NEURO, and NON-CANCER-GI on HEN grew significantly, however, the rates remained with no statistical differences in the observation period (Figure 2; Supplementary Table 4). In total, 72.77% of all patients with PEG were not on HEN.

**FIGURE 3:**
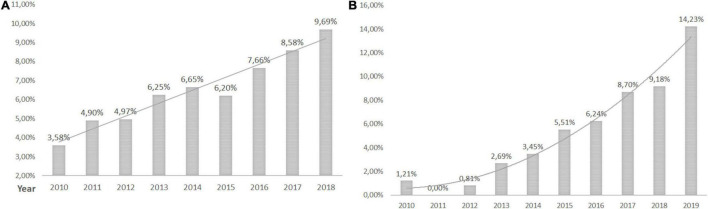
Head and neck cancer patients with PEG. **(A)** The percentage of head and neck cancer patients with gastrostomies in Poland and **(B)** the percentage of head and neck cancer patients with gastrostomies on HEN.

**FIGURE 4:**
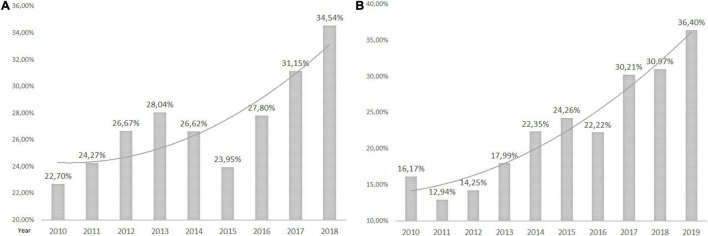
Esophageal cancer patients with gastrostomy. **(A)** The percentage of all esophageal cancer patients with gastrostomies in Poland and **(B)** the percentage of esophageal cancer patients with gastrostomies on HEN.

Of 18,112 patients with CANCER as a primary or secondary disease, 11.86% were in the HEN program. Polish NCR recorded 117,382 HNC and 12,330 patients with esophageal cancer between 2010 and 2018 (12). In total, 6.62% of HNC and 23.97% of esophageal cancer patients with gastrostomies were on HEN during the observation period (Figures 3, 4 and Table 3).

**TABLE 3:** Patients with cancer (primary or secondary diagnosis) with percutaneous endoscopic gastrostomies (PEGs) on home enteral nutrition (HEN).

Year	Cancer (N;%)^1^	Head and neck (N;%)	Respiratory and mediastinum (N;%)	Esophageal (N;%)	Gastric (N;%)	Other GI (N;%)^2^	Other/not specified (N;%)
2010	1051; 8.28%	413; 1.21%	56; 0%	266; 16,17%	165; 16.36%	115; 10.43%	36; 0%
2011	1235; 5.34%	572; 0%	63; 0%	309; 12.94%	147; 10.88%	110; 9.09%	34; 0%
2012	1395; 6.45%	616; 0.81%	51; 0%	379; 14.25%	142; 17.61%	128; 4.69%	67; 0%
2013	1589; 9.13%	782; 2.69%	80; 6.25%	378; 17.99%	154; 26.62%	133; 7.52%	57; 0%
2014	1678; 9.59%	869; 3.45%	97; 10.31%	358; 22.35%	134; 24,63%	141; 5.67%	79; 0%
2015	1700; 10.76%	889; 5.51%	95; 9.47%	338; 24.26%	129; 27.13%	156; 5.13%	80; 0%
2016	2112; 10.65%	1089; 6.24%	122; 4.1%	414; 22.22%	145; 26.9%	223; 9.42%	111; 0%
2017	2198; 14.19%	1173; 8.7%	107; 14.02%	437; 30.21%	141; 34.04%	172; 8.72%	139; 0%
2018	2512; 15.37%	1351; 9.18%	134; 16.42%	507; 30.97%	143; 40.56%	218; 11.47%	130; 0%
2019	2642; 18.66%	1434; 14.23%	129; 9.3%	511; 36.4%	156; 37.18%	255; 12.94%	135; 0%
Overall	18112; 11.86%	9188; 6.62%	934; 8.35%	3897; 23.97%	1456; 26.1%	1651; 8.96%	868; 0%

*Patients with cancer as a primary or secondary diagnosis were included (% – the percentage of patients with cancer on HEN). ^1^All patients with cancer and ^2^Other GI-liver, pancreatic, and intestinal cancer.*

### Regional Differences in the Number of Percutaneous Endoscopic Gastrostomy

**FIGURE 5:**
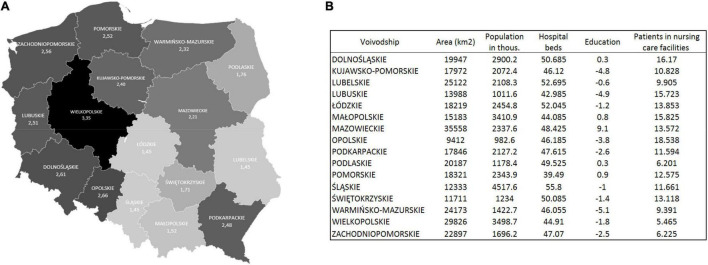
**(A)** The median number of gastrostomies per 10,000 citizens in Poland from 2010 to 2020. **(B)** Voivodeships characteristics. Population data from 2019 (according to the Polish Department of Statistics). Hospital beds and patients in nursing care facilities—median (from 2010 to 2020) per 10,000 citizens. Education (calculated as the difference between the percentage of the population with higher education by voivodeship and the national average).

The median number of PEGs per 10,000 citizens was the highest in Wielkopolskie voivodeship (3.35) and the lowest in Łódzkie (1.44) (Figure 5). PEG prevalence correlated positively with the number of patients in nursing care facilities and negatively with rates of citizens with high education and the number of hospital beds in the region (Table 4). Trend analysis showed statistically significant growths in the number of gastrostomies in all voivodeships (Supplementary Table 2).

**TABLE 4:** Voivodeship regional characteristics.

Variable	Median	IQR	Correlation coefficient	Significance Level P
Physicians^1^	48.28	14.53	−0.03	0.74
Physicians (primary workplace)^2^	22.63	4.27	−0.12	0.13
Hospital beds	47.12	6.22	−0.39	**<0.0001**
Citizens of cities	6031.3	1412.41	−0.01	0.91
Citizens of villages	3968.7	1412.41	0.01	0.91
Highly educated^3^ citizens	−1.55	3.3	−0.25	**<0.0001**
Highly educated^3^ citizens- cities	−2.00	6.1	−0.17	**0.02**
Highly educated^3^ citizens- villages	−0.1	2.6	−0.23	**<0.0001**
Patients in nursing care facilities	11.78	5.14	0.17	**0.03**

*^1^Every workplace, ^2^Active physicians with a license in the region, ^3^The indicator was calculated as the difference between the percentage of the population with higher education (adults) by voivodeship and the national average.*

*Numbers calculated per 10,000 citizens. IQR, interquartile range.*

## Discussion

This study is the first nationwide analysis of PEG tube placement in Poland. A median number of PEG tube insertions per year was 8,413 with a significant (more than 2.5-fold) growth observed from 2010 to 2020. Only a few studies show an up-to-date prevalence of PEG tube placement in other counties. In total, 140,000 PEGs per year were reported in Germany (13) and more than 216,000 in adult patients in the United States (14). Japanese long-term observations showed decreasing numbers of PEG in the years 2007–2015 (15). Since nationwide and long-term observations are difficult to conduct, we found no similar European studies with current data to compare with Polish statistics. Although we observed a growing trend in Poland, the number of PEGs decreased in 2020 by 12.35% in comparison with 2019. Nearly 40% fewer PEG tubes were inserted in the subpopulation of patients with cancer. We assume that it was caused by the limitations in medical procedures during the severe acute respiratory syndrome coronavirus 2 (SARS-CoV-2) pandemic. Data from the United Kingdom confirm that even 20% of endoscopy services were not performing diagnostic examinations in May 2020 due to the pandemic (16). Studies from the United States showed procedures aimed to diagnose cancer (colonoscopies and biopsies) were limited in 2020 and new cancer diagnoses decreased by 13% (17). The following years will show whether this is a temporal occurrence or a more permanent issue in the healthcare system.

All Polish patients with cancer are reported in the NCR (11). Our data indicated an increase in PEG prevalence among patients with cancer in the Polish population. On the other hand, the trend analysis showed that the rate of patients with cancer in the general PEG population decreased from 15.6% (in 2010) to 10% (in 2020). About 6.61% of HNC and 27.46% of patients with esophageal cancer in Poland had PEG. It is known that nearly half of patients with HNC are malnourished (18, 19) and 65% require PEG for 4 weeks and longer (20). In 76% of Scandinavian hospitals, prophylactic PEG is considered routinely, however, only in 2 of 16 of those centers, more than half of the patients had PEG tubes inserted. In 24% of centers, prophylactic PEG was never applied and NGT was a preferred method in 67% of centers (21). Studies show that prophylactic PEG in patients with HNC undergoing chemoradiotherapy is associated with less weight loss and hospital readmission than in the reactive placement group (22). Kapała et al. proved in the Polish study that an organized nutritional care program introduced and supervised by the specialized nutrition team (NST) reduced complication rates, prevented down-dosing of oncological treatment, and weight loss of patients with HNC (23). A growing number of patients with cancer on HEN are observed in European studies. In Poland, 14% in 2013 (7) and 33.9% in 2018 were qualified for HEN due to oncological primary disease (24). Patients with HNC are a dominant group. In the report of the British Association for Parenteral and Enteral Nutrition (BANS), HNC accounted for 77% of new patients with cancer in 2010 and 80% in 2015 (25, 26). In other reports, 11.5–24% of patients on HEN had HNC and 9.8–25% had upper GI cancer (3, 27–29).

In Poland, commercial enteral formulas (FSMP) and the support of specialized NST are reimbursed for patients with gastrostomies during the hospital stay or in the ambulatory setting when qualified for the HEN program (scheme of reimbursement presented in Supplementary Figure 1). The implementation of the HEN program in Poland was proven to be cost-effective and improved clinical outcomes (reduced infectious complications, hospital admissions, and length of stay) (30). Nevertheless, our data showed that 72.4% of all patients with gastrostomies were not under specialized nutritional support and were reimbursed HEN. The rates of cancer patients with PEG on HEN are growing, however, only 18.66% with a primary or secondary oncological diagnosis were on HEN in 2019. About 6.62% of HNC, 23.97% of esophageal cancer, 15.8% with neurological diagnosis, 39.34% with malnutrition and dysphagia, 23.71% of non-cancer GI diseases, and 12.13% of cardio-respiratory and metabolic patients with gastrostomies were on HEN from 2010 to 2019. Temporal funding limitations for HEN reimbursement and lack of knowledge on nutritional support of healthcare professionals might have contributed to those statistics, however, possible reasons for low rates of HEN qualifications need to be analyzed in future studies. Although those data are specific to the local situation and healthcare organization in Poland, this study shows a need to track the patients in the therapeutic pathway and address the weak points of the system to provide proper care for the majority of individuals. Possible problems for countries with no reimbursement of nutritional support may be even more prominent.

A 6.9-fold increase (8.3-fold from 2010 to 2019) and growing rates from 8.8 to 21.4% (of all PEG tube placements) of patients with cardiorespiratory and metabolic diseases were observed. Most of the patients required gastrostomy in the course of cardiac or circulatory failure (33.8%), pneumonia (12.46%), or other respiratory diseases (43.2%). The number of neurological patients increased 5.4 times (6-fold from 2010 to 2019). Interestingly, a low rate of patients with dementia was observed (4.82% of neurological patients). However, this can be explained by the study design limitations. It was based on medical records and ICD-10 codes reported by physicians. The most common primary disease leading to PEG tube placement in our study was malnutrition and dysphagia (37.2% in 2020), which might have been a consequence of other diseases. Gastrostomy use for enteral nutrition in dementia especially in elderly patients is controversial. ESPEN recommends PEG or PEG-PEJ for long-term nutrition, however, comments that NGT may be equally beneficial in many cases (31). A recent Cochrane systematic review showed no clear benefit for PEG vs. NGT for patients with dementia in terms of survival or nutritional status. However, no well-designed randomized trials were available for the analysis (32). We observed that rates of patients with PEG at more advanced ages are increasing (65–74 and over 85 years) and decreasing in younger groups (18–24, 45–54, and 55–64). Trends showing the more advanced age of patients with PEG should be monitored in the future. A discussion on the qualification strategies and future studies on PEG benefits and possible complications in elderly patients are needed. In the Japanese population, half of the patients who qualified for PEG were at least 80 years old (15). In a retrospective study on hospitalized patients, the number of PEGs in the elderly (more than 65 years) increased in the United States from 1993 to 2003 (33). However, more recent data presented decreasing trend in patients with neurodegenerative disorders (34).

Interestingly, significant regional diversity in the prevalence of PEG tube placement in Poland was found in the study. A 2.3-fold difference between the voivodeships with the highest and lowest numbers of PEGs was calculated per 10,000 citizens (1.445 vs. 3.351). Regions with higher numbers of patients in the nursing care facilities had higher numbers of PEG tube placements. This may be coherent with the situation in other countries since it was described that the majority of PEG tubes are placed for patients in nursing homes (13). The PEG prevalence in German nursing homes was 5.6%; 55.3% were inserted before and 44.7% after the admission. The rural vs. urban location of the nursing home was not a differentiating factor, however, the size of the institution and the number of staff influenced PEG prevalence (35). Studies show that number of hospital beds is an important factor influencing hospital logistics and consequently the capacity and access to healthcare services (36). We found that voivodeships with a lower number of hospital beds have higher rates of gastrostomies. Poland has more than an average number of hospital beds among the Organization for Economic Co-operation and Development countries (OECD) with high regional disparities. Most productive regions in Poland develop faster, creating a growing economic gap between the poorest and richest regions. Regional economic disparities are the 6th highest among 29 OECD countries (37). Eastern Poland, especially in rural areas, has a worse self-assessed health status than western and urban (38). However, regional associations with health status may be more complex since many diversities are observed even within voivodeships (39, 40). Economic and racial differences were found to influence the rates of PEG tube placement in other studies (41). Our results showed no relationship between PEG prevalence and the number of citizens in cities and villages. However, higher education was associated with lower numbers of PEG. This may be explained by other studies showing that higher education correlated with better health status and longer life (42). Nevertheless, a broad socio-economic, cultural, and political view is needed to fully understand this topic. Although some correlations were statistically significant in our study, we cannot be sure that we identified the cause of the unequal distribution of PEG procedures in Poland due to the retrospective character of the analysis.

## Conclusion

A growing number of PEG tube placements in Poland was observed. The dominating primary diagnoses of patients qualified for PEG were malnutrition, dysphagia, and cardio-respiratory and metabolic diseases. Despite the reimbursement of the HEN program, nearly two-thirds of patients with PEGs were “outside the system” of specialized nutritional care. What is particularly alarming is that a little more than 1 to 10 patients with PEG were on HEN in the oncological subpopulation. Long-term observation shows that patients are qualified for PEG at a more advanced age. Significant diversity of PEG prevalence was found between administrative regions in Poland. A high number of patients in nursing care facilities, lower education of citizens, and lower number of hospital beds were associated with higher numbers of PEG tube insertions in the region.

## Data Availability Statement

The data are available from the corresponding author upon reasonable request.

## Author Contributions

MF: conceptualization, methodology, formal analysis, investigation, resources, supervision, project administration, and writing—original draft preparation. MF, AW, and KS-Ż: validation. MF, SK, AS-S, and MB: data curation. MF, SK, AS-S, MB, KS-Ż, and JM-S: writing—review and editing. MF and JM-S: visualization. AW and KS-Ż: statistical analysis. All authors have read and agreed to the published version of the manuscript.

## Conflict of Interest

The authors declare that the research was conducted in the absence of any commercial or financial relationships that could be construed as a potential conflict of interest.

## Publisher’s Note

All claims expressed in this article are solely those of the authors and do not necessarily represent those of their affiliated organizations, or those of the publisher, the editors and the reviewers. Any product that may be evaluated in this article, or claim that may be made by its manufacturer, is not guaranteed or endorsed by the publisher.
