# Supplementary material for: Prevalence and Trends in Percutaneous Endoscopic Gastrostomy Placement: Results From a 10-Year, Nationwide Analysis
Source: Front Nutr. 2022 May 30;9:906409. doi: 10.3389/fnut.2022.906409 (PMC9189377; doi:10.3389/fnut.2022.906409)
Supplement: Supplementary file 1 [file Table_1.DOCX]

Supplementary Material

Table 1. Age distribution and trends

| Age | 18–24 | 25–34 | 35–44 | 45–54 | 55–64 | 65–74 | 75–84 | 85+ | All |
| --- | --- | --- | --- | --- | --- | --- | --- | --- | --- |
| Year | N; ratio; % | N; ratio; % | N; ratio; % | N; ratio; % | N; ratio; % | N; ratio; % | N; ratio; % | N; ratio; % | N; ratio |
| 2010 | 90; 1.06; 2.24 | 122; 1.11; 3.04 | 166; 1.11; 4.13 | 518; 1.13; 12.89 | 1064; 1.08; 26.47 | 728; 1.1; 18.11 | 842; 1.07; 20.95 | 489; 1.51; 12.17 | 4019; 1.13 |
| 2011 | 114; 1.16; 2.38 | 135; 1.11; 2.82 | 172; 1.12; 3.59 | 644; 1.07; 13.43 | 1241; 1.07; 25.88 | 936; 1.08; 19.52 | 1023; 1.06; 21.33 | 530; 1.09; 11.05 | 4795; 1.08 |
| 2012 | 171; 1.28; 2.89 | 154; 1.12; 2.6 | 255; 1.09; 4.31 | 651; 1.12; 11 | 1411; 1.07; 23.83 | 1208; 1.09; 20.41 | 1387; 1.09; 23.43 | 683; 1.05; 11.54 | 5920; 1.09 |
| 2013 | 185; 1.18; 2.74 | 161; 1.18; 2.38 | 242; 1.11; 3.58 | 702; 1.1; 10.39 | 1710; 1.1; 25.32 | 1369; 1.1; 20.27 | 1535; 1.1; 22.73 | 850; 1.09; 12.59 | 6754; 1.1 |
| 2014 | 191; 1.39; 2.5 | 182; 1.17; 2.38 | 296; 1.14; 3.88 | 656; 1.08; 8.59 | 1871; 1.11; 24.5 | 1650; 1.07; 21.6 | 1709; 1.08; 22.37 | 1083; 1.08; 14.18 | 7638; 1.09 |
| 2015 | 209; 1.56; 2.48 | 225; 1.13; 2.67 | 333; 1.1; 3.95 | 681; 1.08; 8.09 | 1992; 1.09; 23.66 | 1808; 1.07; 21.47 | 1853; 1.07; 22 | 1320; 1.08; 15.68 | 8421; 1.09; |
| 2016 | 194; 1.44; 2.07 | 211; 1.21; 2.25 | 358; 1.1; 3.81 | 755; 1.05; 8.04 | 2137; 1.11; 22.77 | 2118; 1.09; 22.57 | 2114; 1.08; 22.53 | 1498; 1.06; 15.96 | 9385; 1.09; |
| 2017 | 223; 1.35; 2.22 | 276; 1.14; 2.75 | 390; 1.05; 3.89 | 763; 1.05; 7.6 | 2123; 1.08; 21.16 | 2428; 1.09; 24.2 | 2181; 1.07; 21.74 | 1649; 1.06; 16.44 | 10033; 1.08 |
| 2018 | 227; 1.45; 2.07 | 266; 1.19; 2.42 | 416; 1.09; 3.79 | 889; 1.1; 8.09 | 2326; 1.1; 21.18 | 2722; 1.07; 24.78 | 2303; 1.08; 20.97 | 1835; 1.07; 16.71 | 10984; 1.09 |
| 2019 | 183; 1.26; 1.54 | 238; 1.12; 2.01 | 474; 1.08; 4 | 1054; 1.33; 8.9 | 2352; 1.1; 19.85 | 3100; 1.05; 26.16 | 2532; 1.07; 21.37 | 1915; 1.05; 16.16 | 11848; 1.09 |
| 2020 | 99; 1.1; 0.95 | 356; 1.8; 3.43 | 390; 1.05; 3.76 | 824; 1.06; 7.93 | 1975; 1.04; 19.02 | 2848; 1.05; 27.42 | 2163; 1.13; 20.83 | 1730; 1.17; 16.66 | 10385; 1.1 |
| All | 1886; 1.31; 2.09 | 2326; 1.22; 2.58 | 3492; 1.09; 3.87 | 8137; 1.11; 9.02 | 20202; 1.09; 22.4 | 20915; 1.07; 23.19 | 19642; 1.08; 21.78 | 13582; 1.09; 15.06 | 90182; 1.09 |
| P-value (for n; for %) | 0.29; 0.01 | p<0.001; 0.79 | p<0.001; 0.61 | p<0.001; p<0.00 | p<0.001; p<0.00 | p<0.001; p<0.00 | p<0.001; 0.3 | p<0.001; p<0.00 | p<0.001 |
| Beta (for n; for %) | 5.16; -0.12 | 19.79; -0.01 | 28.81; -0.01 | 37.32; -0.52 | 116.69; -0.72 | 239.85; 0.86 | 155.33; -0.09 | 156.49; 0.62 | 759.45 |
| lower 95%CI (for n; for %) | -5.16; -0.21 | 14.04; -0.1 | 21.81; -0.06 | 20.64; -0.78 | 72.79; -0.87 | 212.29; 0.74 | 117.18; -0.27 | 128.56; 0.42 | 617.78 |
| upper 95%CI (for n; for %) | 15.49; -0.04 | 25.54; 0.08 | 35.81; 0.04 | 54; -0.27 | 160.59; -0.57 | 267.42; 0.99 | 193.48; 0.09 | 184.42; 0.81 | 901.11 |

Ratio- number of GT per patient

Table 2. PEGs on 10 000 citizens. National trend and trends in administrative regions (voivodships).

| Year | 2010 | 2011 | 2012 | 2013 | 2014 | 2015 | 2016 | 2017 | 2018 | 2019 | 2020 | P-value^1^ | Beta | lower 95%CI | upper 95%CI |
| --- | --- | --- | --- | --- | --- | --- | --- | --- | --- | --- | --- | --- | --- | --- | --- |
| DOLNOŚLĄSKIE | 1,519 | 1,485 | 1,996 | 2,216 | 2,506 | 2.606 | 2.876 | 3.211 | 3.344 | 3.486 | 3.002 | p<0.001 | 0.198 | 0.145 | 0.251 |
| KUJAWSKO-POMORSKIE | 1.053 | 1.134 | 1.707 | 1.71 | 2.167 | 2.424 | 2.403 | 2.813 | 2.827 | 3.225 | 2.832 | p<0.001 | 0.21 | 0.164 | 0.256 |
| LUBELSKIE | 0.61 | 0.805 | 0.95 | 1.009 | 1.139 | 1.451 | 1.849 | 1.973 | 2.239 | 3.617 | 2.292 | p<0.001 | 0.238 | 0.149 | 0.327 |
| LUBUSKIE | 1.32 | 1.896 | 1.916 | 2.269 | 2.332 | 2.589 | 2.919 | 3.049 | 2.974 | 3.346 | 2.514 | 0.001 | 0.155 | 0.086 | 0.225 |
| ŁÓDZKIE | 0.73 | 0.91 | 1.099 | 1.211 | 1.288 | 1.445 | 1.527 | 1.633 | 2.04 | 2.057 | 1.821 | p<0.001 | 0.127 | 0.1 | 0.153 |
| MAŁOPOLSKIE | 0.742 | 0.817 | 0.934 | 1.016 | 1.382 | 1.525 | 1.925 | 1.946 | 2.238 | 2.496 | 2.244 | p<0.001 | 0.187 | 0.155 | 0.218 |
| MAZOWIECKIE | 1.084 | 1.303 | 1.678 | 1.824 | 2.043 | 2.206 | 2.404 | 2.559 | 2.741 | 2.933 | 2.31 | p<0.001 | 0.161 | 0.107 | 0.214 |
| OPOLSKIE | 1.541 | 2.038 | 2.183 | 2.443 | 2.663 | 2.793 | 3.499 | 3.582 | 3.634 | 3.434 | 2.641 | 0.003 | 0.169 | 0.073 | 0.264 |
| PODKARPACKIE | 1.88 | 1.49 | 1.677 | 2.058 | 2.213 | 2.477 | 2.769 | 2.829 | 3.147 | 3.319 | 3.01 | p<0.001 | 0.177 | 0.131 | 0.223 |
| PODLASKIE | 0.432 | 0.616 | 0.834 | 1.254 | 1.483 | 1.756 | 2.088 | 2.135 | 2.291 | 2.696 | 2.176 | p<0.001 | 0.216 | 0.167 | 0.266 |
| POMORSKIE | 1.29 | 1.645 | 2.147 | 2.085 | 2.379 | 2.517 | 2.864 | 3.229 | 3.239 | 3.687 | 3.328 | p<0.001 | 0.222 | 0.181 | 0.263 |
| ŚLĄSKIE | 0.677 | 0.873 | 0.987 | 1.075 | 1.313 | 1.448 | 1.65 | 1.713 | 1.934 | 2.166 | 2.034 | p<0.001 | 0.149 | 0.133 | 0.166 |
| ŚWIĘTOKRZYSKIE | 0.833 | 1.031 | 1.223 | 1.118 | 1.509 | 1.714 | 1.817 | 2.096 | 2.572 | 2.877 | 2.496 | p<0.001 | 0.2 | 0.16 | 0.241 |
| WARMIŃSKO-MAZURSKIE | 0.983 | 1.459 | 1.419 | 1.968 | 2.013 | 2.579 | 2.316 | 2.648 | 2.879 | 3.001 | 2.316 | p<0.001 | 0.172 | 0.102 | 0.241 |
| WIELKOPOLSKIE | 1.197 | 1.681 | 2.255 | 2.991 | 3.274 | 3.564 | 4.1 | 4.178 | 5.042 | 4.451 | 3.351 | 0.001 | 0.304 | 0.161 | 0.446 |
| ZACHODNIOPOMORSKIE | 1.183 | 1.497 | 1.91 | 2.413 | 2.392 | 2.556 | 2.616 | 2.953 | 2.736 | 3.227 | 6.213 | 0.002 | 0.326 | 0.156 | 0.496 |
| **POLAND** | 1.043 | 1.243 | 1.536 | 1.754 | 1.984 | 2.188 | 2.441 | 2.61 | 2.855 | 3.079 | 2.704 | p<0.001 | 0.198 | 0.161 | 0.235 |

1. Trend analysis p-value


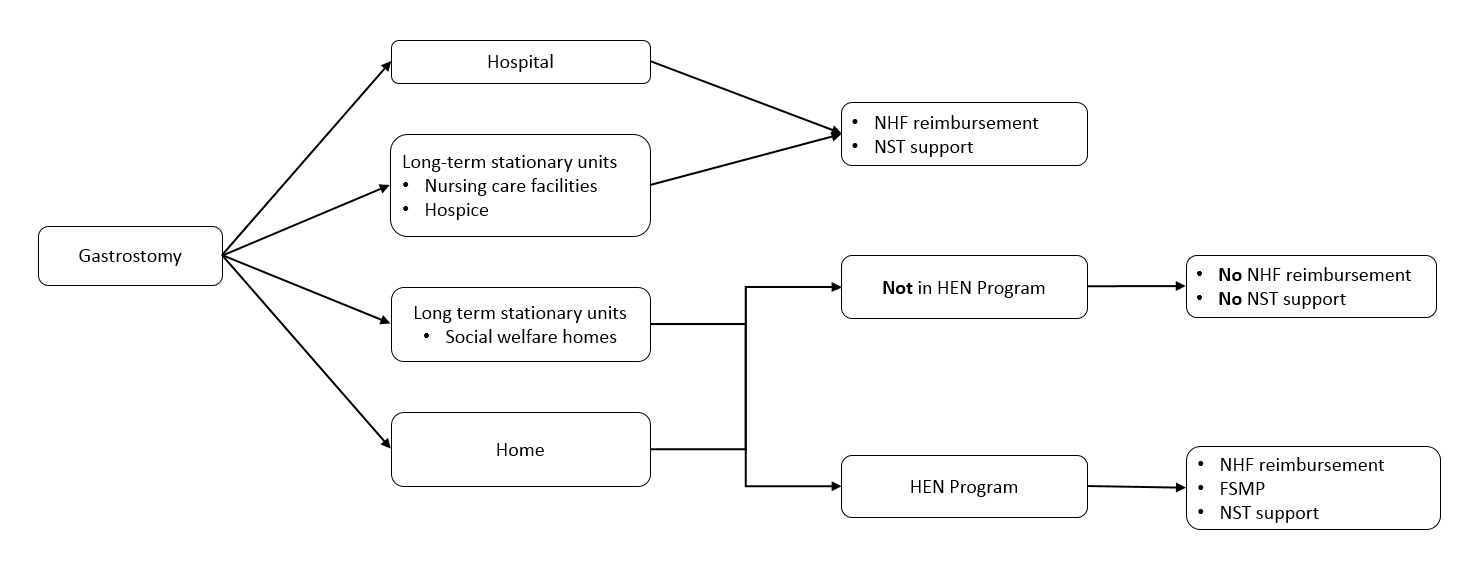


Figure 1. Reimbursement of enteral nutrition for patients with gastrostomy in Poland

NST-Nutrition support team, FSMP- Food for special medical purposes

Table 3. Primary diagnosis and trends

| Year | Non-Cancer GI (N; %) | Other (N; %) | Cardio-resp. & metabolic (N; %) | Neurology (N; %) | Malnutrition/Dysphagia (N; %) | Cancer (N; %) | All (N) |
| --- | --- | --- | --- | --- | --- | --- | --- |
| 2010 | 574; 16.8% | 733; 21.5% | 299; 8.8% | 244; 7.2% | 1027; 30.1% | 533; 15.6% | 3410 |
| 2011 | 559; 13% | 662; 15.4% | 465; 10.8% | 403; 9.4% | 1648; 38.4% | 553; 12.9% | 4290 |
| 2012 | 533; 10.1% | 790; 15% | 737; 14% | 464; 8.8% | 2151; 40.7% | 604; 11.4% | 5279 |
| 2013 | 556; 9.2% | 791; 13.1% | 978; 16.2% | 606; 10.1% | 2460; 40.9% | 630; 10.5% | 6021 |
| 2014 | 563; 8.2% | 834; 12.1% | 1287; 18.7% | 664; 9.6% | 2920; 42.4% | 618; 9% | 6886 |
| 2015 | 602; 7.9% | 920; 12% | 1528; 20% | 830; 10.8% | 3122; 40.8% | 651; 8.5% | 7653 |
| 2016 | 640; 7.5% | 943; 11.1% | 1760; 20.7% | 923; 10.8% | 3500; 41.1% | 749; 8.8% | 8515 |
| 2017 | 635; 6.9% | 1018; 11.1% | 1967; 21.5% | 1117; 12.2% | 3614; 39.4% | 817; 8.9% | 9168 |
| 2018 | 599; 6% | 954; 9.6% | 2219; 22.3% | 1174; 11.8% | 4084; 41% | 940; 9.4% | 9970 |
| 2019 | 584; 5.4% | 1048; 9.8% | 2477; 23.1% | 1358; 12.6% | 4196; 39.1% | 1081; 10.1% | 10744 |
| 2020 | 449; 4.7% | 1352; 14% | 2071; 21.5% | 1221; 12.6% | 3593; 37.2% | 969; 10% | 9655 |
| P-value (for n; for %) | 0.88; p<0.001 | p<0.001; 0.01 | p<0.001; p<0.001 | p<0.001; p<0.00 | p<0.001; 0.35 | p<0.001; 0.02 |  |
| Beta (for n; for %) | -0.84; -0.99 | 51.76; -0.74 | 216.41; 1.36 | 110.15; 0.5 | 288.26; 0.31 | 52.77; -0.44 |  |
| lower 95%CI (for n; for %) | -12.75; -1.31 | 33.93; -1.27 | 177.03; 0.96 | 95.33; 0.38 | 210.99; -0.41 | 38.47; -0.8 |  |
| upper 95%CI (for n; for %) | 11.08; -0.67 | 69.6; -0.21 | 255.78; 1.76 | 124.96; 0.62 | 365.54; 1.04 | 67.08; -0.08 |  |

Table 4. Patients with gastrostomies on HEN (primary diagnosis)

| Year | Non-Cancer GI (N;%) | Other (N;%) | Cardio-resp. & metabolic (N;%) | Neurology (N;%) | Malnutrition/Dysphagia (N;%) | Cancer (N;%) |
| --- | --- | --- | --- | --- | --- | --- |
| 2010 | 131; 23% | 140; 19% | 33; 11% | 39; 16% | 265; 26% | 87; 16% |
| 2011 | 120; 21% | 128; 19% | 36; 8% | 39; 10% | 402; 24% | 66; 12% |
| 2012 | 104; 20% | 159; 20% | 69; 9% | 79; 17% | 534; 25% | 90; 15% |
| 2013 | 149; 27% | 158; 20% | 86; 9% | 94; 16% | 759; 31% | 145; 23% |
| 2014 | 126; 22% | 200; 24% | 159; 12% | 90; 14% | 1008; 35% | 161; 26% |
| 2015 | 121; 20% | 255; 28% | 192; 13% | 141; 17% | 1221; 39% | 183; 28% |
| 2016 | 155; 24% | 230; 24% | 240; 14% | 154; 17% | 1524; 44% | 225; 30% |
| 2017 | 167; 26% | 296; 29% | 233; 12% | 165; 15% | 1613; 45% | 312; 38% |
| 2018 | 175; 29% | 247; 26% | 295; 13% | 203; 17% | 1819; 45% | 386; 41% |
| 2019 | 138; 24% | 311; 30% | 321; 13% | 226; 17% | 2155; 51% | 493; 46% |
| P-value (for n; for %) | 0.05; 0.13 | p<0.001; p<0.001 | p<0.001; 0.01 | p<0.001; 0.21 | p<0.001; p<0.001 | p<0.001; p<0.001 |
| Beta (for n; for %) | 4.7; 0.52 | 20.17; 1.23 | 34.67; 0.5 | 21.16; 0.33 | 211.1; 3.11 | 44.04; 3.68 |
| lower 95%CI (for n; for %) | -0.01; -0.19 | 13.95; 0.76 | 30.08; 0.14 | 18.21; -0.23 | 195.16; 2.51 | 31.63; 2.96 |
| upper 95%CI (for n; for %) | 9.41; 1.23 | 26.39; 1.7 | 39.25; 0.87 | 24.12; 0.9 | 227.04; 3.7 | 56.44; 4.4 |
